# High spatial resolution identification of hematoma in inhomogeneous head phantom using broadband fNIR system

**DOI:** 10.1186/s12938-018-0605-2

**Published:** 2018-11-27

**Authors:** E. Sultan, A. H. Gandjbakhche, K. Pourrezaei, A. S. Daryoush

**Affiliations:** 1 Dept. of Electronics Engineering, College of Technological Studies, PAAET, Kuwait, Kuwait; 2 Dept. of ECE, Drexel University, Philadelphia, PA 19104 USA; 3 Eunice Kennedy Shriver National Institute of Child Health and Human Development, National Institutes of Health, Bethesda, MD 20892 USA; 4 School of Biomedical Engineering, Science, and Health Systems, Drexel University, Philadelphia, PA 19104 USA

**Keywords:** fNIR, TBI, FEM, DE, COMSOL, Optical transmitter Tx, Optical receiver Rx, Tri-wavelength VCSEL, PDW, IL, IP

## Abstract

This paper presents a novel method for early detection of hematomas using highly sensitive optical fNIR imaging methods based on broadband photon migration. The NIR experimental measurements of inhomogeneous multi-layer phantoms representing human head are compared to 3D numerical modeling over broadband frequencies of 30–1000 MHz. A finite element method (FEM) simulation of the head phantom are compared to measurements of insertion loss and phase using custom-designed broadband free space optical transmitter (Tx) and receiver (Rx) modules that are developed for photon migration at wavelengths of 670 nm, 795 nm, 850 nm, though results of 670 nm are discussed here. Standard error is used to compute error between 3D FEM modeling and experimental measurements by fitting experimental data to the $a\sqrt {frequency} + b$. Error results are shown at narrowband and broadband frequency modulation in order to have confidence in 3D numerical modeling. A novel method is established here to identify presence of hematoma based on first and second derivatives of changes in insertion loss and phase (∆IL and ∆IP), where frequency modulated photons sensitive to different sizes of hematoma is identified for wavelength of 670 nm. The high accuracy of this comparison provides confidence in optical bio-imaging and its eventual application to TBI detection.

## Introduction

Bio-imaging techniques have shown a great deal of confidence in improving the diagnosis and clinical care for civilians and military personnel, who experience a traumatic brain injury (TBI) due to sports, auto accidents, or explosions from improvised explosive device (IED) [1]. Injuries of this caliber can lead to temporary or permanent impaired brain functionality. Commonly categorized as either an open or closed injuries; scalp and skull penetration due to a sharp object/bullet are seen in open, while closed injuries occur when the head is shaken violently due to acoustic boom. Both can lead to mild to severe TBI, causing sheared or torn brain axons, blood vessels, or both [2]. Most undiagnosed TBI injuries are closed head injures and without fractures. Another type of injury to the brain is the development of a hematoma. Disrupted blood vessels that leak into the layers of dura mater or between the skull and dura mater, might lead to epidural or subdural hematomas [3]. It can be medically confirmed by CT scan or MRI imaging techniques [4]. Due to lack of mobility and high cost of these imaging modalities, optical imaging using functional near infrared (fNIR) is being developed by understanding the relation between migrated photons and biological media.

Photons in the near-IR (NIR) region are particles that have both absorption and scattering properties when interacted with biological media such as tissue, cells, or organs [5]. Biological media have optical properties that include both reflection and absorption characteristics [5]. Photons suffer broadening and eventual decay as it travels because of multiple scattering and absorption when penetrated and traveled through bio-media. Propagation of these photons within bio-media depends on the media optical properties of absorption and scattering which is defined by particles within cells, cell organelles, and fiber structure [6]. Optical parameters of the tissue depend on both absorption and scattering of photons which reveals information related to hemoglobin and water concentration [7]. This information is used to determine an important aspect of biological functionality that is related to concentration of oxygen/de-oxygenated level in hemoglobin and the amount of water when photons have penetrated and traveled in bio-media and this method of imaging is called functional near infrared (fNIR) imaging.

The fNIR based spectroscopic studies of biological media have shown the capability of measuring the absorption and scattering sensitivity of water, oxygenated, and de-oxygenated hemoglobin at different wavelengths. In that regard any impact that will lead to disorder the normal functionally of the neurons of brain could be then registered by disorder in the absorption of oxygenated or de-oxygenated hemoglobin using diffused photon NIR (DPNIR) [8]. The location and percentage of oxygen absorbed in the brain can be related to different physiological activities through fNIR system. Measuring the reduction in de-oxygenated hemoglobin or increase in oxygenated hemoglobin leads to an increase in oxygenated blood volume and having this localized information related to hematoma detection [9], would aid in earlier diagnosis. Resulting is faster, more efficient and cost-effective treatment. Moreover, early detection and efficacy of this proposed treatment could be compared using fNIR imaging, by monitoring the performance of human brain regions over time.

**Fig. 1:**
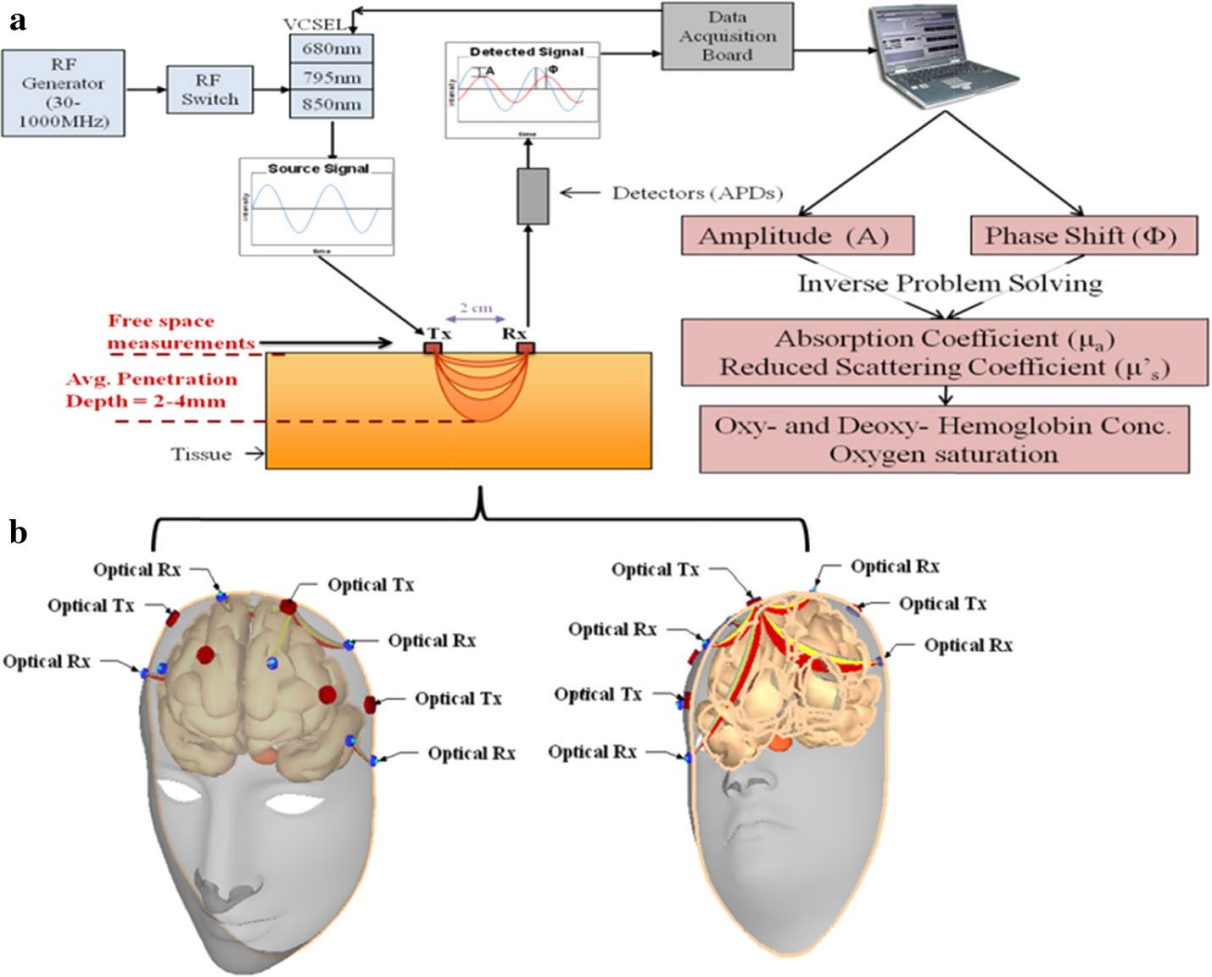
Conceptual representation of a high spatial resolution system fNIR optical imaging: **a** broadband frequency modulated multi-wavelength fNIR system using insertion loss (IL) and insertion phase (IP) measurements, and **b** photon migration from optical transmitter (Tx) to various optical receiver (Rx) through a human head at various modulation frequencies

This paper will examine for the first time the ability of a broadband free-space fNIR in detecting high optical absorbing media that resembles a human head with an embedded hematoma and studies the spatial resolution detection of a hematoma. The concept followed in this paper is based on the assumption that a hematoma is generated after a blast or accident, and that broadband fNIR system is advantageous in detecting smaller hematomas. Focusing mainly on hematomas, the method of detection consists of both numerical modeling and an experimental measurement that follows the frequency modulated photon concept traveling in multi-layer inhomogeneous human brain phantoms. It should be noted that these phantoms resembles a human head, which is embedded with a hematoma at an outer layer of the cortex. A conceptual representation of human head phantom and optical system setup is shown in Fig. 1. Optical transmitter (Tx) and receivers (Rx) are strategically located on head phantom across the hematoma. This study is part of early detection of traumatic brain injury (TBI) using a helmet mounted functional near infrared (fNIR) device [10, 11]. A 3D FEM modeling of diffusion equation (DE) for inhomogeneous head phantoms is employed to quantify hematoma sizes approaching sub-centimeter spatial resolutions. First, this paper presents comparison between 3D FEM and experimental result at wavelength of 670 nm to quantify accuracy of model fitting by comparing insertion loss and phase from 3D modeling and measurements results that are curve fitted over various modulation frequency bandwidths. Second, a signal processing method is proposed by taking advantage of first and second derivative of the differential measurements of IL between cortex with and without occlusion for detection hematoma as photons interact with different sizes of occlusion representing hematoma.

## Numerical modeling and experimental methods

**Fig. 2:**
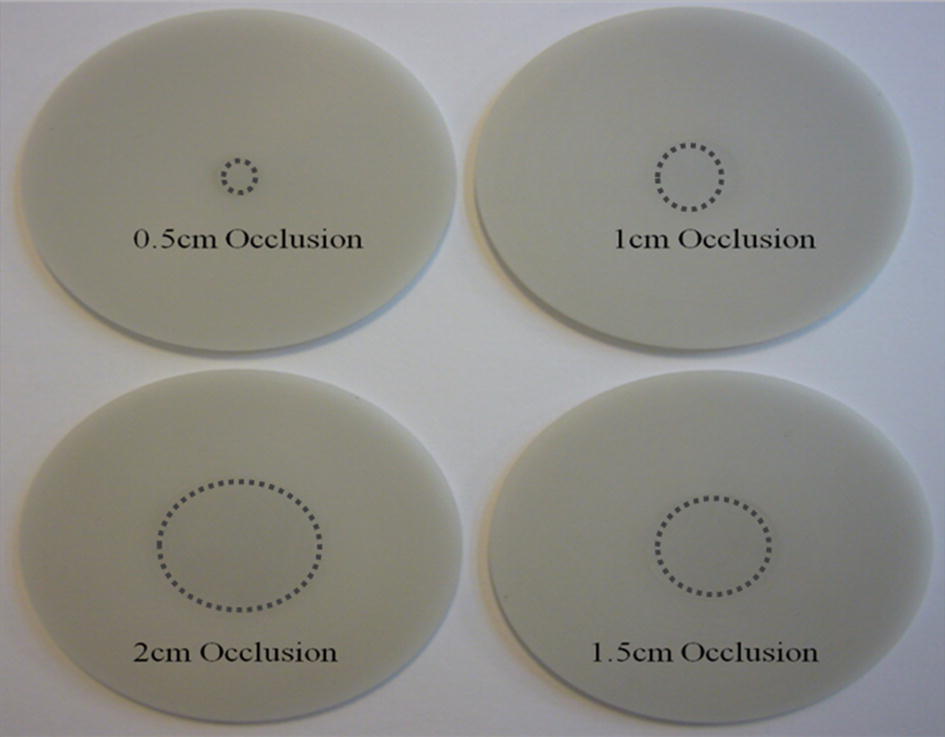
Images of solid phantoms of different sizes of occlusion embedded in cortex phantom to resemble formation of different sizes of hematoma in cortex. The dashed circles emphasize optical absorption difference between cortex and hematoma

**Fig. 3:**
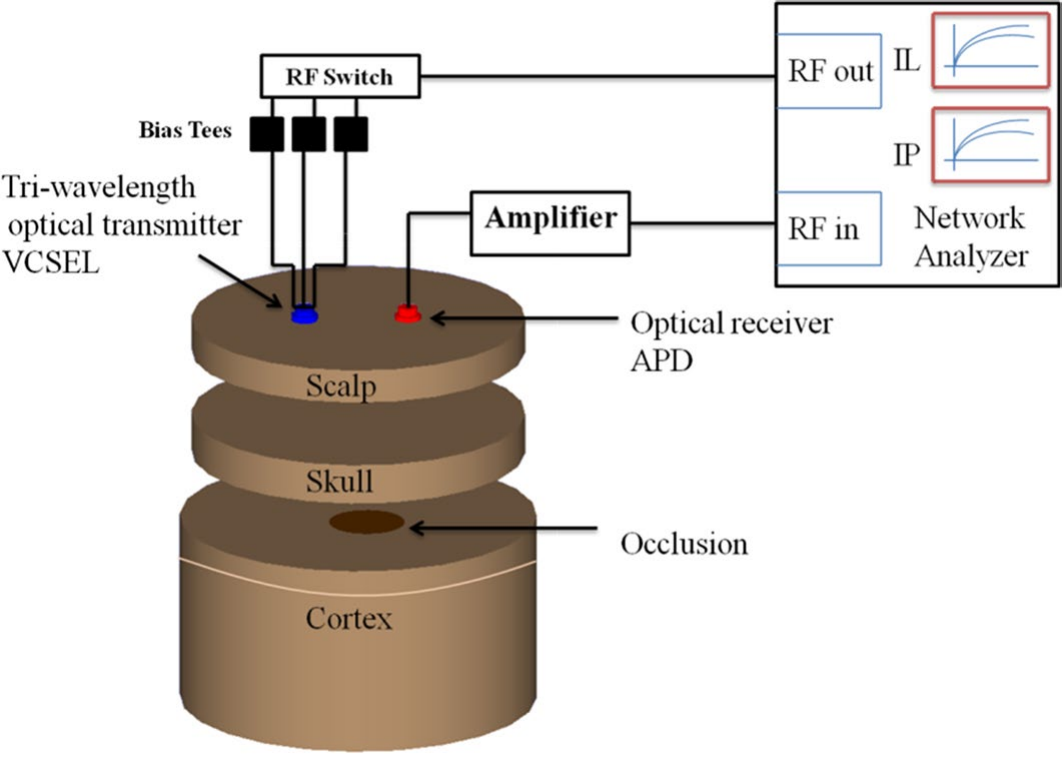
Optical system and three layer phantoms with embedded occlusion

Broadband frequency modulation method has proven high efficiency when applied to functional optical imaging systems on homogenous and inhomogeneous biological media, and proven to present with high optical parameter extraction accuracy as shown in previous publications [12, 13]. The method is to be extended here to monitor the early formalization of hematoma as a sign of TBI. An optical system using broadband modulation is used along with three layers of phantom, embedded with different sizes of occlusion. After an injury, a hematoma can develop slowly, in relation to time. Therefore an occlusion of different sizes in diameter is used for this study to measure the sensitivity of our numerical model and experimental performance in detecting smaller sizes of an occlusion. These occlusions have optical properties similar to the one formed after a blast or concussion. The phantoms used in the experiments with various occlusions are depicted in Fig. 2, where different sizes of occlusion are designed and embedded in the center of the cortex layer; the optical properties of the occlusions [14, 15] are shown in Table 1. Occlusions are designed to start with a diameter of 0.5 cm and go up to 2 cm, with increments of 0.5 cm, with a height of 0.2 cm. The three layers with the embedded occlusions are then stacked on top of each other and the functional NIR imaging based on broadband frequency modulation is performed at three different wavelengths with a separation of 2 cm, as shown in Fig. 3.

**Table 1:** Phantom optical parameter properties at wavelength of 670 nm

	Diameter (cm)	Thickness (cm)	$\mu_{a}$ (cm^−1^)	$\mu_{s}^{'}$ (cm^−1^)
Cortex	5	3.5	0.35	15
Scalp	5	0.3	0.15	13
Skull	5	0.4	0.12	8
Occlusion 1	0.5	0.2	0.6	18
Occlusion 2	1	0.2	0.6	18
Occlusion 3	1.5	0.2	0.6	18
Occlusion 4	2	0.2	0.6	18

Accurate 3D FEM numerical modeling requires experimental verification of change in amplitude and phase of the incident photon wave that is related to absorption and scattering of the biological media is presented in the frequency domain as insertion loss (IL) and insertion phase (IP). Whereas IL is change in amplitude and IP is change in phase of RF signal modulating NIR photons as they pass through inhomogeneous turbid media. Finite element modeling (FEM) of DE using the commercially available software (multi-physics COMSOL from FEMLab) is preferred over Monte-Carlo simulation of photons because of its good accuracy at a much shorter processing time. The 3D FEM simulation provides graphical or tabular values of DE solution for a given geometry and boundary conditions [13]. The next section will explain the tools used to model the human head phantom with embedded occlusion that replicates TBI condition.

### Finite element method

The solution of the diffusion equation is modeled in COMSOL through Helmholtz equation for FE numerical analysis [13], where the general form of the DE is:

1 $$ \nabla \left( { - D\nabla \emptyset \left( {r,t} \right)} \right) + \mu_{a} \emptyset \left( {r,t} \right) = S\left( {r,t} \right) $$

where $\emptyset \left( {r,t} \right)$ is the photon flux, $D = \frac{1}{{3\left( {\mu_{a} + \mu_{s}^{'} } \right)}}$, and in a frequency modulated NIR system $S\left( {r,t} \right) = S_{O} \left( {1 + me^{j\omega t} } \right)$ where *m* is amplitude modulation index at time harmonic modulation frequency of $\omega .$ COMSOL representation of the Helmholtz equation is:

2 $$ - \nabla \cdot \left( {{\text{c}}\nabla {\text{u}}} \right) + au = f $$

**Fig. 4:**
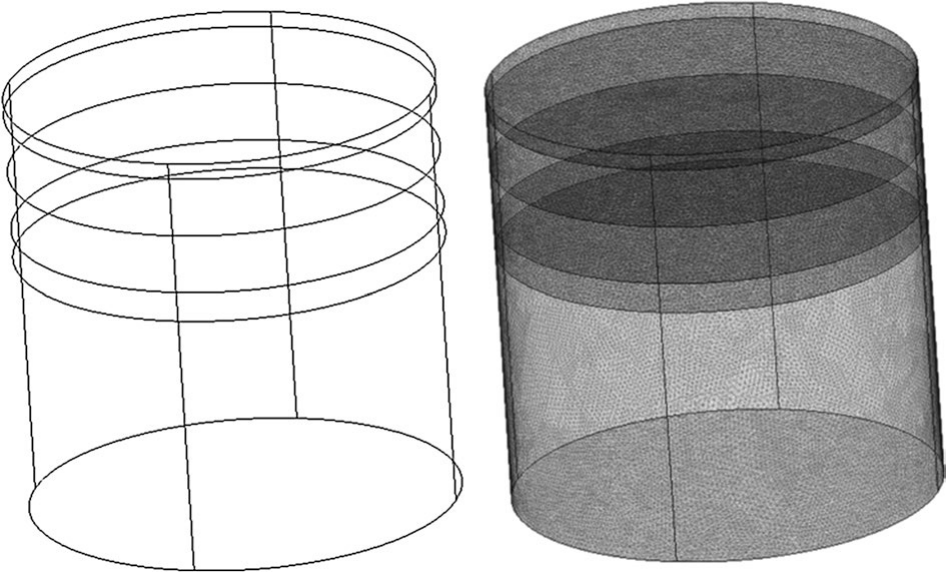
3D human head layers and the mesh structure applied to simulation

Diffusion parameters of inhomogeneous layers are listed in Table 2. It has been in our experience that having mesh structures (cf. Fig. 4) of element sizes of 80 nm and 1000 nm in regions around optical Tx/Rx and everywhere else respectively would predict an accurate result (e.g., error of less than 5%) of amplitude and phase change. A typical computation time of 4 s for single 2D simulation and 26 s for single 3D simulation is experienced using an Intel Core i3 processor PC. These meshing element sizes are also recommended for interfaces (cf. Fig. 4), when multi-layer human head phantoms are modeled [16]. In COMSOL, the boundary condition is set either through Dirichlet or Neumann boundary conditions, where they are set to express the fluence rate, *u*, at desired boundaries. Dirichlet and Neumann boundary conditions are chosen appropriately for air-dielectric (or dielectric–dielectric) and radiation condition in region surrounding phantom. The mathematical representation is expressed [17] as:

3 $$ n \cdot (\left( {c\nabla u} \right)_{1} - \left( {\left( {c\nabla u} \right)_{2} } \right) + q \cdot u = g $$

4 $$ h \cdot u = r $$

where $h, r, q, \;{\text{and}}\; g$ are the boundary coefficients for the phantom modeling. For the diffused photon flux, h = 1 and r = 0. For the matching boundary of air dielectric and dielectric and dielectric interfaces q = 0 and g = 0.

**Table 2:** Coefficients and relevant expressions used in Helmholtz sub-domains that govern each layer of human head phantom

	Coefficient “c”	Coefficient “a”
Layer 1	$\frac{1}{{3\left( {\mu_{a1} + \mu_{s1}^{'} } \right)}}$	$\mu_{a1}$
Layer 2	$\frac{1}{{3\left( {\mu_{a2} + \mu_{s2}^{'} } \right)}}$	$\mu_{a2}$
Layer 3	$\frac{1}{{3\left( {\mu_{a3} + \mu_{s3}^{'} } \right)}}$	$\mu_{a3}$
Occlusion	$\frac{1}{{3\left( {\mu_{a4} + \mu_{s4}^{'} } \right)}}$	$\mu_{a4}$

### Experimental measurement procedures

The principle of transmitting modulated photons and detecting the traveled photons requires an optical transmitter and optical receiver that operate in a linear region for the desired modulated broadband frequency [12]. The monitoring methods of brain functionality are defined by monitoring different absorption and scattering rates of the oxygenated and de-oxygenated hemoglobin at various wavelengths; therefore, optical transmitters at certain wavelength are desired to quantify insertion loss and phase at various wavelengths to perform inverse problem solution and identify the existence of hematomas in certain regions of the head. The wavelength specifies monitoring the de-oxygenated hemoglobin in the range of 640 to 770 nm [18] while the oxygenated hemoglobin is in the range of 830–920 nm [18]. The range of 640 to 770 nm results in high absorption of photons in the deoxygenated hemoglobin, which means a source of photons in that range will be required to monitor the deoxygenated hemoglobin. While the range of 830 to 920 nm will result in a high photon absorption in the oxygenated hemoglobin, meaning a source of photons in that range will be required to monitor the oxygenated hemoglobin.

The overall measurement system consists of Automatic Network Analyzer (Anritsu MS4623B) as an RF source and sensitive RF receiver and broadband optical transmitter various wavelengths and optical receiver modules. The ANA acts as a highly sensitive frequency modulator and demodulator instrument, while the optical transmitter and receiver modules act as broadband modulated photon sources and highly sensitive (i.e., low intensity noise) optical detector. Optical transmitters consisting of tri-wavelengths at 670 nm, 850 nm, and 795 nm, are used in this study to monitor peak absorption and cross over absorption of the deoxygenated and oxygenated hemoglobin, respectively. The frequency modulation of the tri-wavelength photon source is controlled by an SP3T RF switch (Hittite HMC245QS16) to drive three high power vertical cavity surface emitting lasers (VCSELs) (670 nm, 795 nm, 850 nm). A separate DC biasing control and RF modulating signals is provided for each wavelength through lowpass–highpass Bias-T networks. The VCSEL diode laser (Vixar; Module V3WLM) based optical sources emit high power (output power of about 5 mW) Gaussian optical beams with low astigmatism at threshold currents of 8.5 mA for the 670 nm, 9.5 mA for the 795 nm, and 2.8 mA for the 850 nm. A printed circuit board has been designed and fabricated using a commercial substrate (FR4), to accommodate all surface mount technology (SMT) components. In absence of PIN Photodiode based commercial high gain optical receivers, APD (Hamamatsu ADP module C5658) have been used along with a built-in trans-impedance amplifier [12, 13].

## Broadband frequency modulation experimental and simulation results

**Fig. 5:**
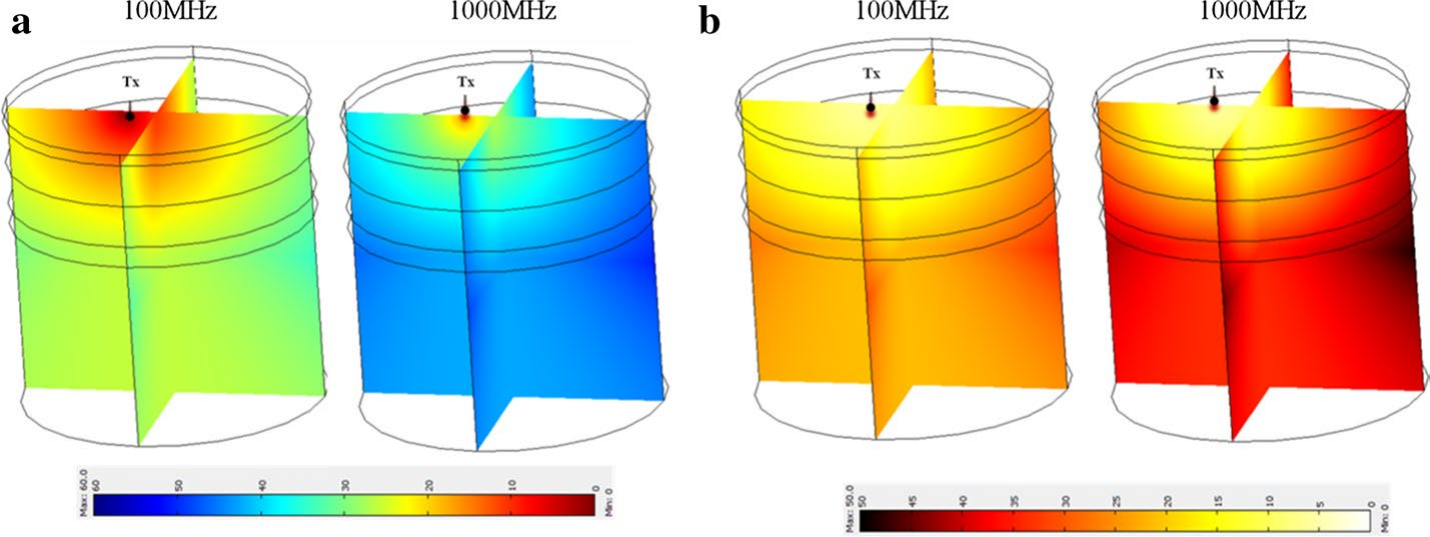
3D numerical simulation of IL (**a**) and IP (**b**) at two frequencies of 100 MHz and 1000 MHz for four layer human head phantom without any hematoma occlusion as control

**Fig. 6:**
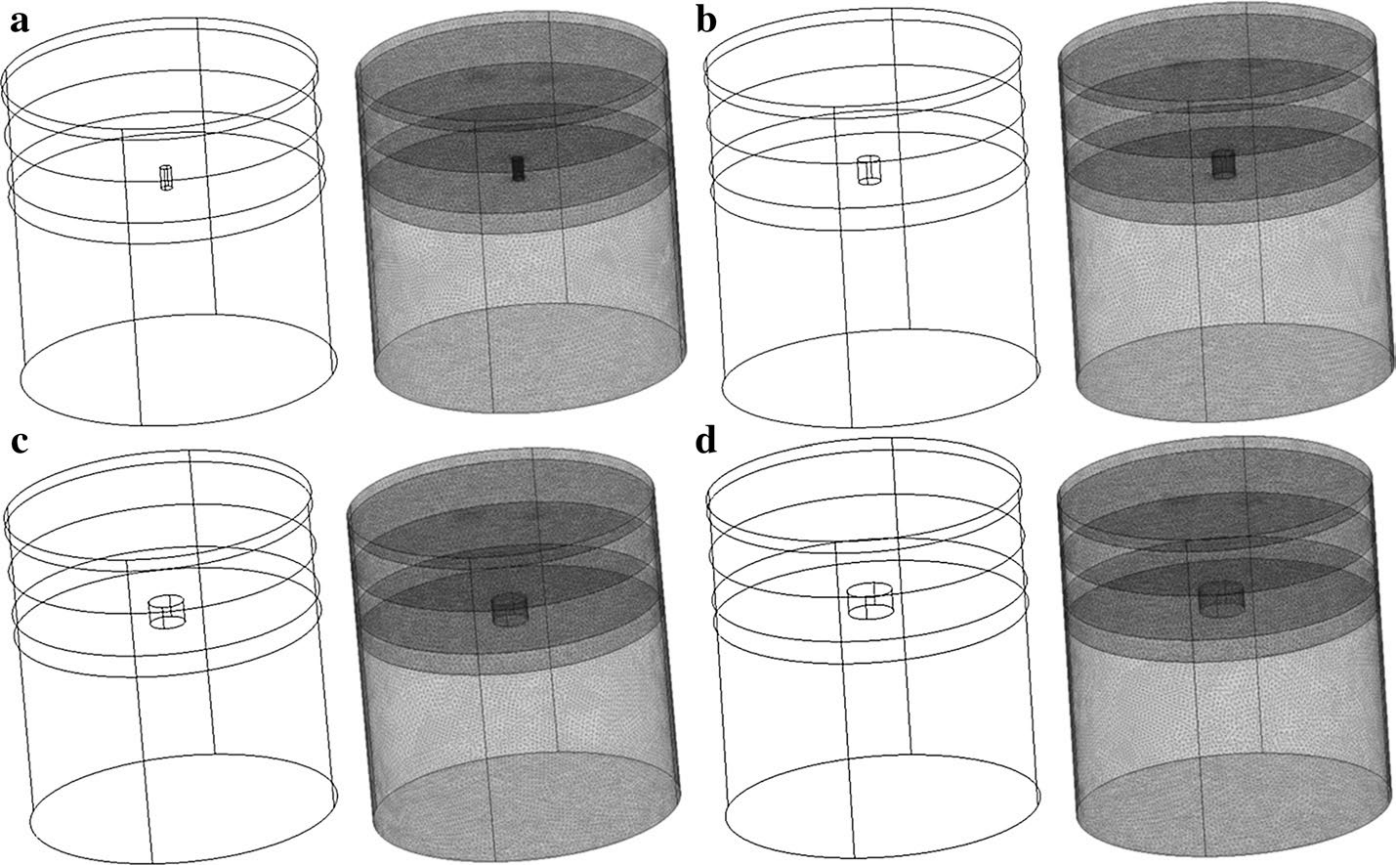
3D human head layers with 0.5 cm (**a**), 1.0 cm (**b**), 1.5 cm (**c**), 2.0 cm (**d**) in diameters of embedded occlusions and the mesh structures applied to simulation for compromise in high accuracy and short simulation times

Simulated result by COMSOL is done using broadband frequency modulation from 30 to 1000 MHz. Figure 4 shows the 3D concept and mesh structure of four layers of phantoms stacked up to form a head phantom with different optical absorption and scattering parameters without any occlusions. The first top two layers are scalp and skull layer, while the last layer is pure cortex layer and these layers have homogenous optical parameters. However, the third layer is a layer of cortex, which could be without any occlusion (i.e., a homogenous optical parameters), as depicted in Fig. 4 or with capability of adding different sized occlusions to resemble a formation of a hematoma in the cortex with inhomogeneous optical parameters (cf. Fig. 6). Identifying the impact of occlusion requires comparison of layers with (occlusion 1–4) against the layer without occlusion (i.e., control). 3D numerical simulation for without occlusion (i.e., control) case is performed at two frequencies of 100 MHz and 1000 MHz and shown in Fig. 5, while experimentally this comparison could be made that as both Tx and Rx are pushed away from the occlusion region using translation stages (cf. Figure 2 of reference [13]). These results are later used for identification of high optical activities of occlusions with high absorber.

**Fig. 7:**
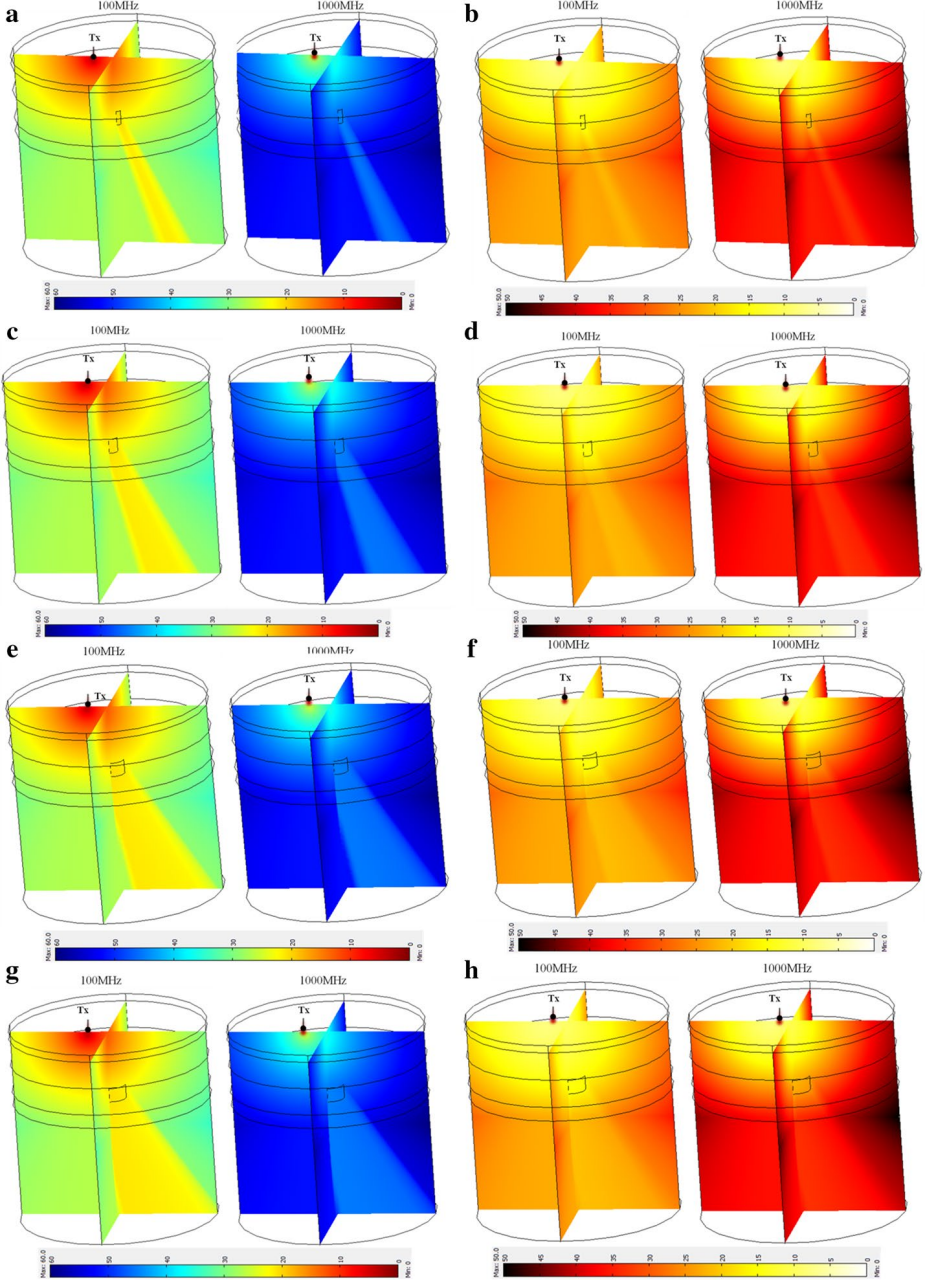
3D FEM simulation of IL and IP at two frequencies of 100 MHz and 1000 MHz for different sizes of occlusions embedded in the third layer of a four layer human head phantom; **a** IL and **b** IP for 0.5 cm occlusion; **c** IL and **d** IP for 1 cm occlusion; **e** IL and **f** IP for 1.5 cm occlusion; **g** IL and **h** IP for 2 cm occlusion

**Fig. 8:**
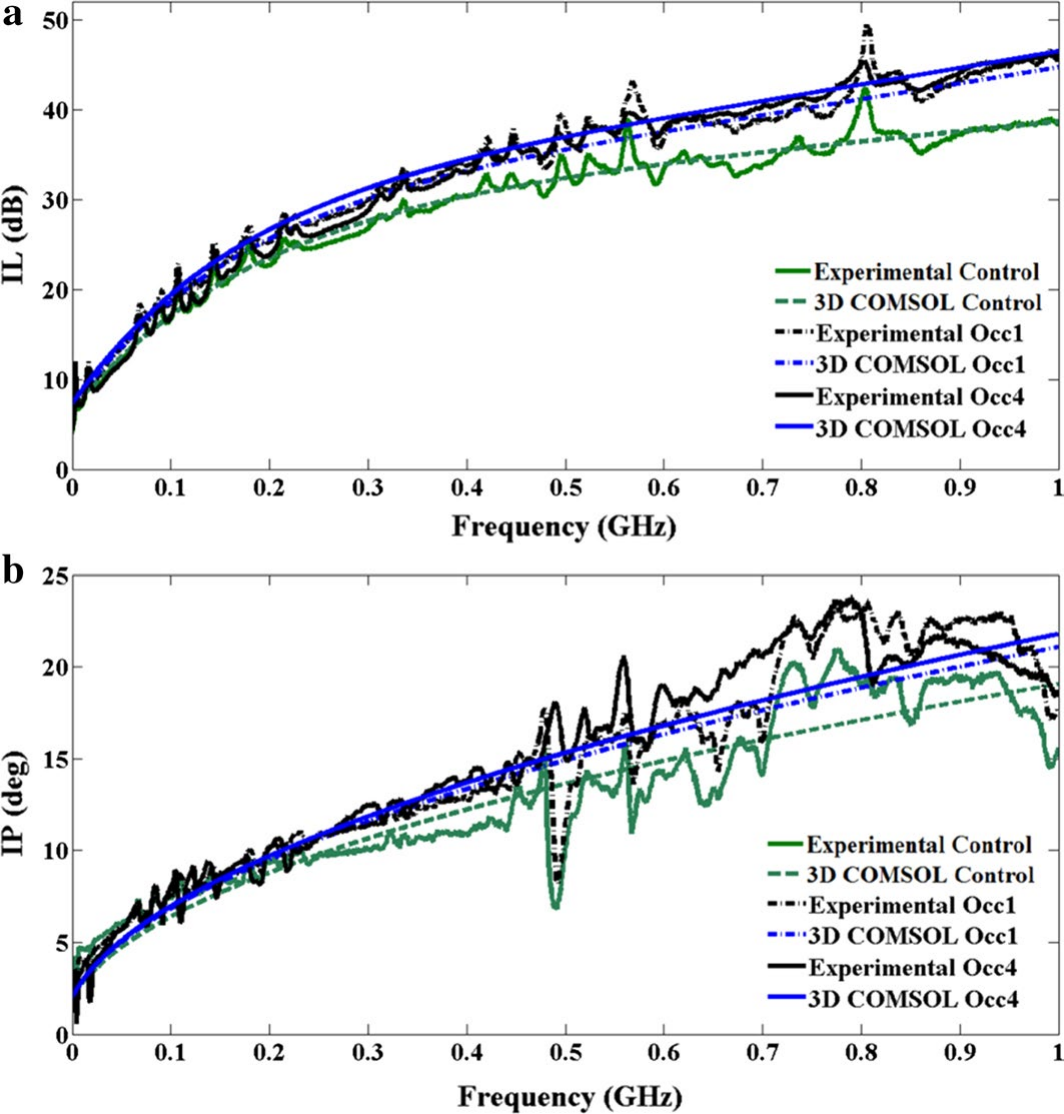
Comparison of measured and FEM simulation results at NIR of 670 nm: **a** IL, **b** IP from 30 to 1000 MHz

Different sized occlusions embedded in the third layer of the head phantom are numerically simulated based on mesh structure shown in Fig. 6. Simulations at two frequencies of 100 MHz and 1000 MHz for different sizes of occlusions are shown in Fig. 7, while experimentally broadband measurements for different occlusion sizes and numerical simulation is shown in Fig. 8. Insertion loss and insertion phase measurements at an optical transmitter and receiver separation of 2 cm around the embedded occlusion, center of the phantoms, is performed along with null location. The null location is considered a control location that is used to compare against change of IL and IP when different sized occlusions are present within the cortex. Experimentally, measured results are done at wavelengths of 670 nm because of the available information of known phantom optical parameters that was provided from the manufacturer.

At wavelengths of 670 nm, it is observed that the behavior for both IL and IP follows mathematical formulation of $a\sqrt {frequency} + b$ as expected due to the solution of diffusion equation derived for photons traveling in bio-media [12]. From these results, it is comprehended that the occlusion effect is dominating IL at frequencies greater than 300 MHz, while it is not clear at lower frequencies. The location of the occlusion is in the shallow layers of the cortex and because the photon path at high frequency modulation is interacting with the occlusion, the effect of the high absorbing occlusion shows at a frequency-stamp around 300 MHz. The important observation is the sensitivity observed by Insertion loss and insertion phase for the smallest and largest occlusions, and that is shown when at 1000 MHz of frequency modulation the difference between control and the smallest occlusion is 6–7 dB of IL and 4–5° of IP for the 670 nm wavelength. This shows that for a small occlusion, the broadband frequency modulation system has an advantage of high sensitivity for detecting high oxygenated and de-oxygenated activities. In order to compare the experimental result to the numerical result a method using standard error calculation is used. The standard error, shown in Table 3, is based on integrated differences between the simulated FEM results for the multilayer phantom with occlusion and the curve fitted experimental results to the mathematical formulation of $a\sqrt {frequency} + b$ for each frequency data point divided by number of frequency data points. Mathematically the standard error is expressed as:

**Table 3:** Error between curved fitted experimental data and COMSOL 3D over various bandwidths

Wavelength	Third layer w/occlusion 1 Error %		Third layer w/occlusion 2 Error %		Third layer w/occlusion 3 Error %		Third layer w/occlusion 4 Error %	
	IL	IP	IL	IP	IL	IP	IL	IP
670 nm								
30–1000 MHz	2.26	3.35	2.16	3.27	2.47	3.35	2.35	3.35
30–300 MHz	3.45	4.09	3.37	4.19	3.22	4.09	3.26	4.13
300–500 MHz	5.85	5.35	5.44	5.42	5.64	5.35	5.63	5.68
500–1000 MHz	4.74	4.65	4.24	4.49	4.09	4.74	4.51	4.07

5 $$ standard \;error = \sqrt {\frac{{\mathop \sum \nolimits_{i = 1}^{n} \left( {Curve \;fitted\; experimental \;data - 3D \;FEM\;\varvec{ }simulation} \right)^{2} }}{n}} $$

The low standard error values builds confidence in the 3D FEM modeling of inhomogeneous phantoms and facilitates the avenue to solve the inverse problem for accurate extraction of optical parameters that is required for tomographic imaging.

### Early detection of TBI

**Fig. 9:**
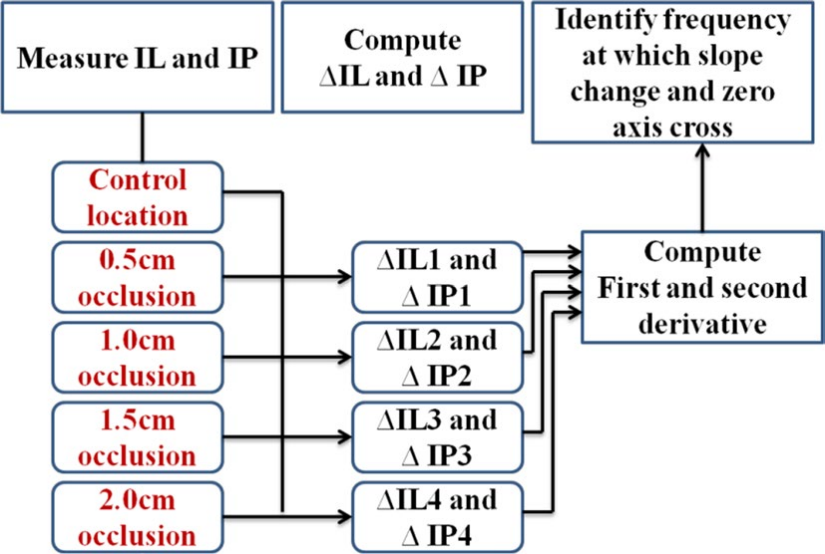
Method of threshold detection based on first and second derivatives of ∆IL and ∆IP

**Fig. 10:**
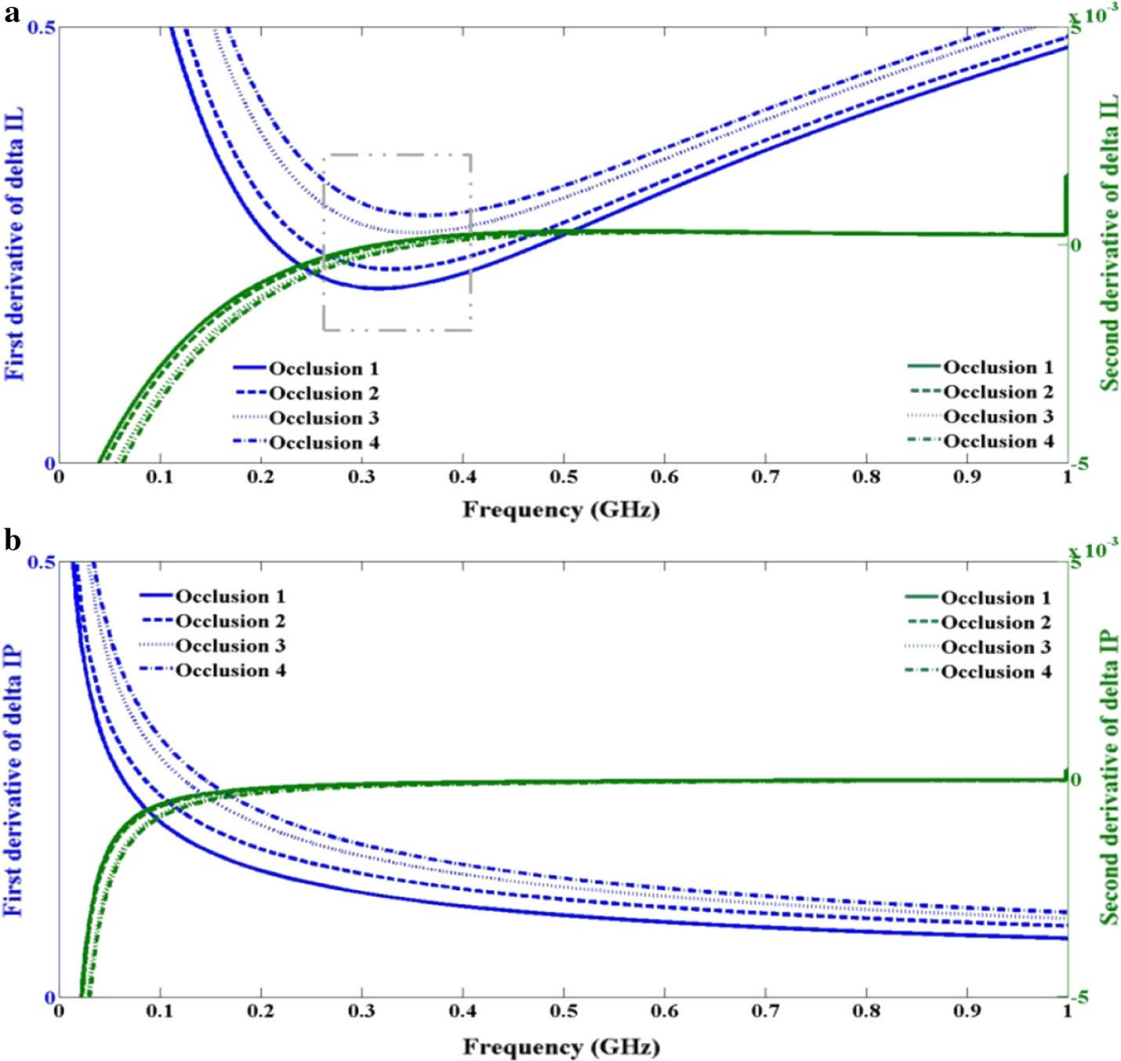
First and second derivatives of curve fitted experimental data of: **a** 670 nm ∆IL, **b** 670 nm ∆IP

Results from previous sections support the task of accurate modeling of TBI using 3D FEM, and as an indication of this process it is observed that a change of slope of the resulted IL occurs when compared to the control IL. This indication is related to the understanding of frequency modulation concept of photons and flight of travel through the inhomogeneous media. Looking at Fig. 8 we see that at frequency around 300 MHz we have a significant change in IL as compared to the surrounding frequencies. We are attributing this change in the sloe of IL to strong absorption of the photos by the occlusion. We are taking the advantage of this fact for identifying a hematoma occlusion. It should be noted that this specific frequency depends on the size and the location of the hematoma occlusion. Knowing the exact modulated frequency at which the most favorable path of photon is interacting with the embedded occlusion is of great interest, and it can be done using a threshold method of identifying a frequency-stamp at which the slope of IL is changed. It is important to understand that change of slope is observed in IL but it’s not clearly observed in IP and that is adjustable due to the close values of $\mu_{s}^{'}$ for stacked layers of scalp, skull, and cortex. The slope of IP is increasingly high without change in slope and that is due to the high values of $\mu_{s}^{'}$. The threshold process is explained in Fig. 9, where first we compute ∆IL between insertion loss due an embedded occlusion and when there is no occlusion. Then taking the first and second derivative of the resulted ∆IL and locating the local minimum and zero frequency-axis cross over as the point of interaction between modulated photon and occlusion. Figure 10 shows the first and second derivative of ∆IL and ∆IP for wavelength of 670 and highlighted region shows the frequency-stamp at which the interaction is taking place. These frequency stamps are related to frequency modulated photon interaction with different sizes of occlusion as shown in Table 4.

**Table 4:** Curve fitted experimental data threshold and frequency-stamp identification for different sizes of TBI

Wavelength	Occlusion size (cm)	Experimental	FEM modeling	Error %
		Freq. of MFP (MHz)	Freq. of MFP (MHz)	Difference between experimental and modeling
670 nm	0.5	376	371	1.3
	1.0	361	355	1.8
	1.5	343	337	1.8
	2.0	330	322	2.5

## Discussions and conclusions

**Fig. 11:**
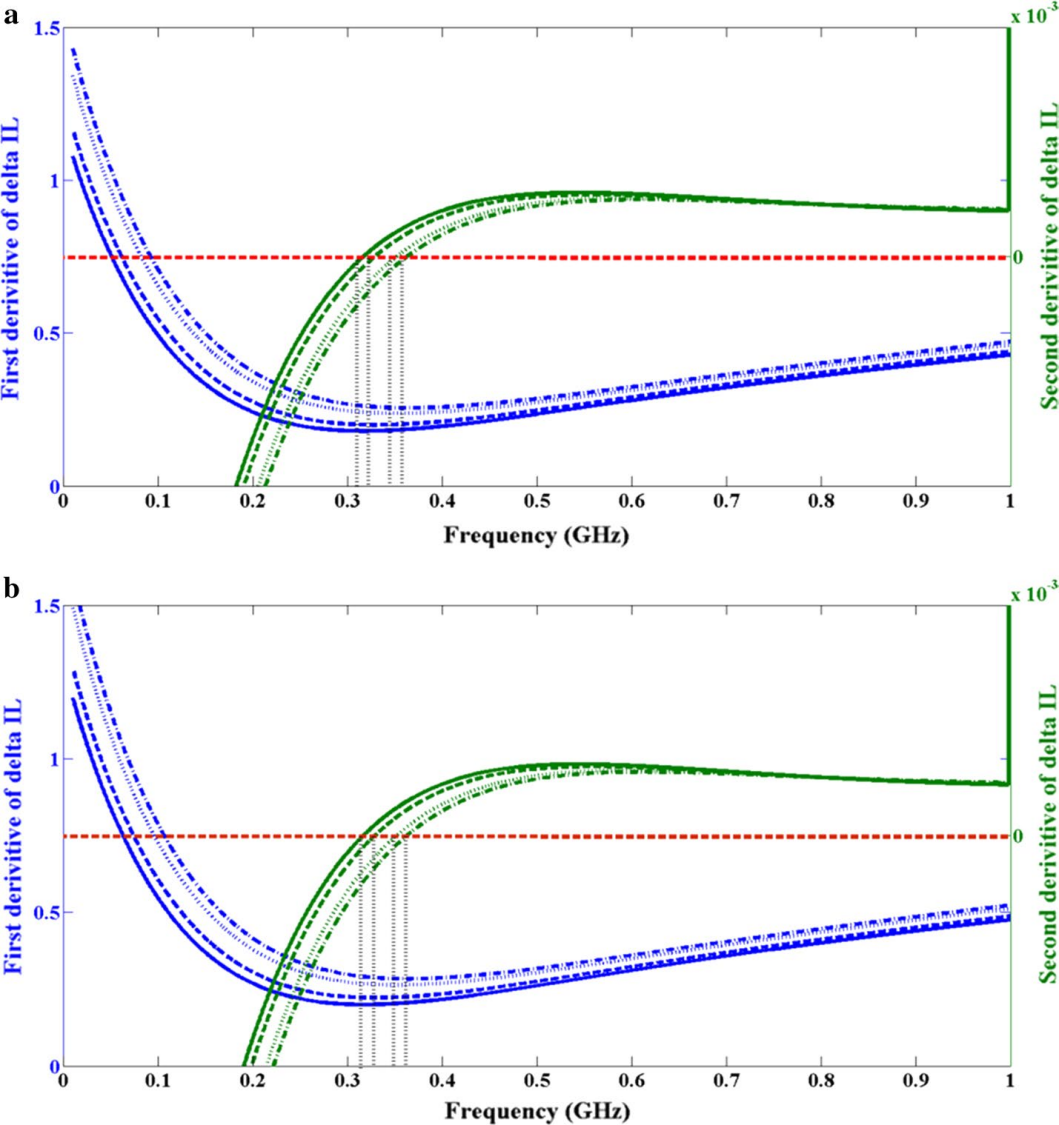
First and second derivatives of ∆IL between control and inhomogeneous third layer with different sizes of occlusions for **a** FEM simulation, and **b** curve fitted experimental data

This paper presents two important aspects of broadband frequency modulated fNIR system in terms of inverse problem of optical parameter extraction of multi-layer and inhomogeneous phantoms. We have demonstrated for the first time numerical modeling in 3D using FEM and its accuracy compared to the actual experimental results of phantom based human brain activity for with (abnormal) and without (normal) hematoma, and its ability to successfully identify early signs of phantom based hematoma formation using highly sensitive signal processing methods. The accuracy of 3D numerical modeling is computed through the standard error calculation between numerical and curve fitted experimental result for wavelength of 670 nm. A high confidence is developed with an error of less than 4%, as rendered in Table 3, between simulation and measurement results over broadband measurements of 30–1000 MHz. The sensitivity of detecting different sizes of hematoma is clear with measurements for curve fitted experimental data and FEM predictions at different frequency-stamps at 670 nm wavelength, as shown in Table 4. FEM modeling shows a great deal of accuracy with modulated photons interacting with the smallest occlusion at 321 MHz and a slope increase of 15–18 MHz for each 0.5 cm increment in occlusion diameter. While curve fitted experimental data shows modulated photons interacting with the smallest occlusion optimally at 330 MHz and frequency increments of 13–19 MHz for each 0.5 cm increment in diameter. These results are illustrated in Fig. 11, which based on the signal processing method described in previous sections, shows the frequency-stamp of each modulated photon interaction with occlusions for both curve fitted experimental data and FEM simulation results.

**Fig. 12:**
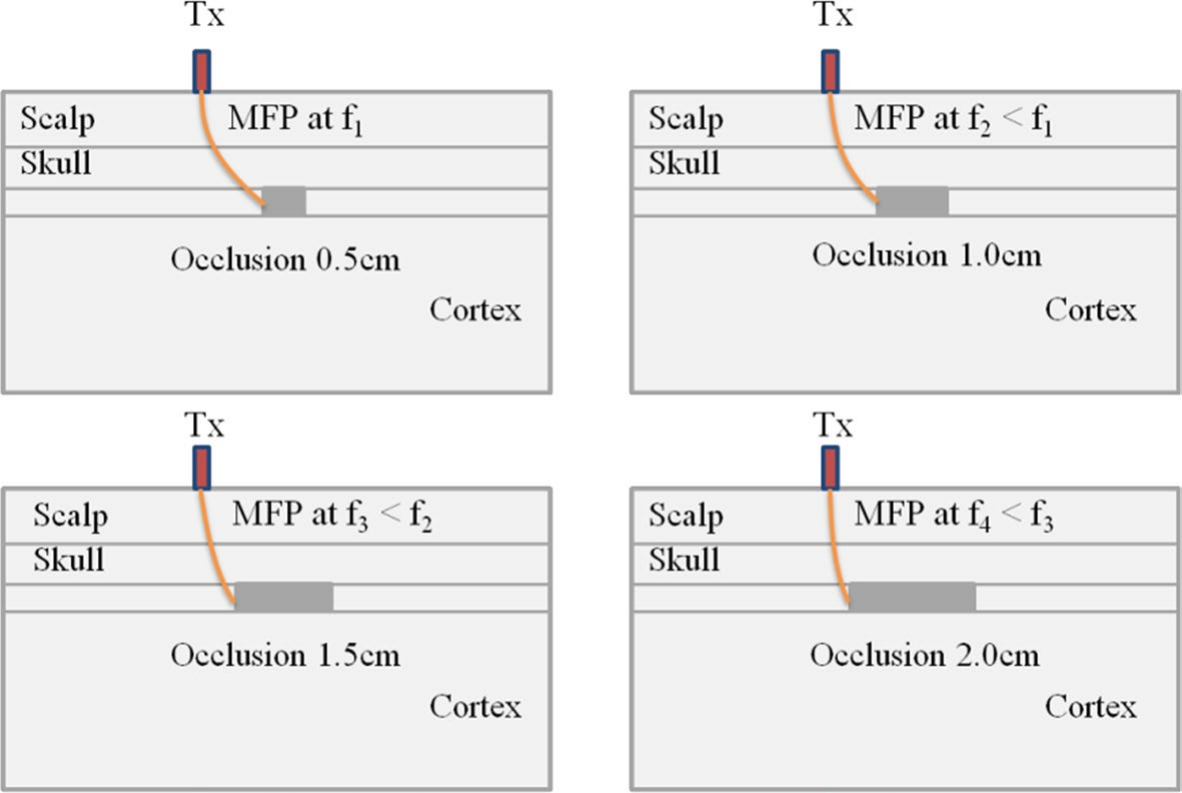
Conceptual results of embedded occlusions and most favorable path for different modulating frequency of diffused photons

Extracted information of frequency stamp of the most favorable path of photons (MFP) gives insight to understanding the behavior of modulated photons in complex bio-media and supports the advantages of using broadband measurements. Frequency-stamp for both experimental and FEM simulation results are rendered in Table 4, and can be used to construct a conceptual modulated photon traveling in multi-layer phantom media as shown in Fig. 12. In fact, for different sizes of occlusions modulated wave of photons movement could be identified for a particular modulating frequency of diffused photons.

In conclusion, frequency modulated fNIR system shows a great potential in early detection of hematomas in multilayer brain phantom and have been validated as a high accurate tool through the use of numerical modeling. This was accomplished using 3D FEM and experimental measurements of IL and IP using broadband photon modulation on cases of normal and abnormal human head phantoms. Numerical modeling of 3D FEM shows a great deal of accuracy associated with homogenous [13] and inhomogeneous biological media [19], and can be used as a tool to perform inverse problem solving because of its low time consumption. Note this concept of 3D numerical modeling could be applied for any complex inhomogeneity of head structure and brain matter. First and second derivative for ∆IL is reported here as a novel method of bio-marker to identify small size of hematoma with high accuracy.

Since this study is performed though one separation of 2 cm between an optical transmitter and receiver, it is important to indicate that the frequency associated with penetration depth and hematoma phantom location is not uniform to different optical transmitter and receiver separation and further adjustment is needed for different measurement topologies. Detection of small sized occlusions resembling formation of a hematoma and structure of each layer of the scalp and skull could be accomplished by taking advantage of the most favorable path of photon dependence on the modulating frequency of photons. This behavior is understood through the use of 3D FEM modeling and accuracy with the experimental measurements, as demonstrated for the first time in this paper. Frequency broadband measurements of IL and IP based on photon migration through multilayer human head phantom shows a promising breakthrough in detecting any high optical activity within certain depth beneath scalp.
